# Another way to measure balanced time perspective: Development and validation of the Time Perspective Inventory

**DOI:** 10.1002/pchj.725

**Published:** 2024-02-01

**Authors:** Xiaobao Li, Houchao Lyu, Zena R. Mello

**Affiliations:** ^1^ Faculty of Education Henan University Kaifeng China; ^2^ Time Psychology Research Center Southwest University Chongqing China; ^3^ China Community Psychology Service and Research Center Southwest University Chongqing China; ^4^ Department of Psychology San Francisco State University San Francisco California USA

**Keywords:** anxiety, life satisfaction, negative affect, positive affect, time perspective inventory

## Abstract

The existing scales for measuring balanced time perspective (BTP) have limitations, such as poor‐fitting structures or a complex calculation method. Based on previous studies, we conceptualize BTP as an individual's overall positive outlook on the past and future and mindfully living in the present. The present study aimed to develop the Time Perspective Inventory (TPI) for Chinese adults, determine its psychometric properties, and examine a simple calculation method for generating a score to represent BTP. In study 1, a 7‐factor structure of TPI – Past Positive, Past Negative, Mindful Present, Present Hedonistic, Present Excessively Task‐oriented, Future Positive, and Future Negative – was established through exploratory (sample 1, *N* = 529) and confirmatory (sample 2, *N* = 577) factor analyses. Findings supported the internal consistency, test–retest reliability, and criterion‐related validity of the TPI. We proposed using the score difference between positive time perspectives and negative time perspectives to calculate the BTP. Findings showed that the correlations between BTP and subjective well‐being indicators and anxiety were higher than among individual dimensions of TPI. Study 2 (sample 3, *N* = 713) compared the effects of the TPI and the Chinese version of the Zimbardo Time Perspective Inventory (ZTPI‐C) on well‐being indicators and anxiety. Results indicated that the variance of life satisfaction, positive affect, negative affect, and anxiety explained by BTP measured with TPI was higher than deviation from BTP (DBTP) measured with ZTPI‐C. Together, the TPI yields reliable and valid BTP scores among Chinese adults.

## INTRODUCTION

Psychological time is a necessary mental framework for individuals to organize and interpret life events. As a fundamental dimension of psychological time, time perspective (TP) refers to a relatively stable characteristic reflecting how individuals view their past, present, and future (Zimbardo & Boyd, 1999). Zimbardo and Boyd (1999) distinguished five common TPs: (1) Past Negative (PN), reflecting a pessimistic and negative attitude towards the past; (2) Past Positive (PP), representing a positive and joyful outlook of the past; (3) Future (F), describing a general orientation to plan; (4) Present Hedonistic (PH), involving a preference for immediate gratification and spontaneity, and a dislike towards planning; (5) Present Fatalistic (PF), describing a helpless and fatalistic attitude towards the future (Zimbardo & Boyd, 1999). Each TP is found to be associated with human functioning such as emotional intelligence, self‐identity, self‐esteem, anxiety, depression, and life satisfaction (Boniwell et al., 2010; Laghi et al., 2013; Stolarski et al., 2011, 2020; Zimbardo & Boyd, 1999).

However, the overuse or underuse of one temporal category may cause dysfunction (Boniwell & Zimbardo, 2004). For instance, F is conducive to academic achievement and planning behaviors, but a completely future‐oriented person may have high time pressure and cannot enjoy any personal indulgence (Zimbardo & Boyd, 1999). High emphasis on PH could cause irresponsible behaviors, such as substance abuse and risky driving (Chavarria et al., 2015; Zimbardo et al., 1997). Thus, Zimbardo and Boyd (1999) further proposed the idea of balanced time perspective (BTP). BTP was initially defined as the mental ability to switch effectively among different TPs based on situational demands (Zimbardo & Boyd, 1999). Considering that the switching capacity is hard to operationalize, Zimbardo and Boyd (2008) redefine BTP as a combination of low scores on PN and PF and relatively high scores on PP, PH, and F; almost all the Zimbardo Time Perspective Inventory (ZTPI)‐based indicators of BTP were calculated with the combination of high adaptive TPs and low non‐adaptive TPs, such as cut‐off point (Drake et al., 2008), cluster‐analysis approach (Boniwell et al., 2010), and the formula of deviation from BTP (DBTP) (Stolarski et al., 2011). BTP is regarded as the focus of positive psychology and is put forward to optimize individuals' social functioning and obtain greater subjective well‐being (Boniwell & Zimbardo, 2004). Thus, subjective well‐being‐related indicators are analyzed as the most natural outcomes of BTP. A meta‐analysis has shown that BTP explains about 40% of subjective well‐being variance (Stolarski et al., 2020), indicating the pronounced effects of temporal balance on people's happy life and mental health.

The most frequently used tool for operationalizing a BTP is the ZTPI, although the ZTPI does have limitations. First, many items in ZTPI are assessing constructs such as fatalism, impulsivity, and conscientiousness rather than TPs (Webster, 2011; Worrell et al., 2018). For instance, “I do things impulsively” is a PH item, but it measures impulsivity. Various separable constructs being measured on one scale could cause poor psychometric properties. For example, the reliability coefficient of PF stays below the minimum threshold in many cultural backgrounds (McKay et al., 2015; Worrell et al., 2018), and replication of the five‐factor model of ZTPI has been problematic with poor model fit indices (Davis & Cernas Ortiz, 2017; Mohammed & Marhefka, 2020). Second, there is controversy about the structure of ZTPI because it does not cover all necessary types of TPs, such as a negative future and a clearly positive present perspective (Carelli et al., 2015; Sobol‐Kwapińska & Jankowski, 2016), which are related to one's temporal balance, subjective well‐being, and depression (Horwitz et al., 2017; Sobol‐Kwapińska & Jankowski, 2016; Vowinckel et al., 2015).

An increasingly used method to calculate BTP is the DBTP formula, but it also has problems (Stolarski et al., 2020). The DBTP formula measures one's fit to the optimal levels of TPs:

$$\text{DBTP}=\sqrt{{\left(\mathrm{oPN}-\mathrm{ePN}\right)}^{2}+{\left(\mathrm{oPP}-\mathrm{ePP}\right)}^{2}+{\left(\mathrm{oPF}-\mathrm{ePF}\right)}^{2}+{\left(\mathrm{oPH}-\mathrm{ePH}\right)}^{2}+{\left(\text{oF}-\mathrm{eF}\right)}^{2}}$$

where ePN, ePP, ePF, ePH, and eF represent empirical scores obtained by individuals in the ZTPI, while oPN, oPP, oPF, oPH, and oF are optimal levels for these TPs and the same for every individual. The DBTP formula assesses the combination of low adaptive TPs and high non‐adaptive TPs, which is not consistent with the initial definition of BTP, that is, the switching capacity among different TPs (Zimbardo & Boyd, 1999). This is not a limitation specific to DBTP but all BTP indicators measured with ZTPI (Stolarski et al., 2020). The more direct problem of DBTP is defining the optimal levels of TPs, which could lead to a paradoxical conclusion (Jankowski et al., 2020). Based on ZTPI, using a 1–5 scoring scale, the optimal scores representing a balanced profile amounted to 3.67 for PP, 3.69 for F, 4.33 for PH, 2.1 for PN, and 1.67 for PF, respectively (Zimbardo & Boyd, 2012). However, the question becomes whether an individual who scores 1 on PN is less balanced than someone who scores 2.1 under the condition that PN has been regarded as maladaptive. Considering that DBTP seems to assume quadratic relations, Jankowski et al. (2020) revised the DBTP formula by using extreme values as the optimal value for most TPs.

### Measuring BTP in the present study

According to most studies in the field (Sobol‐Kwapińska & Jankowski, 2016; Vowinckel et al., 2015; Webster, 2011; Zimbardo & Boyd, 2008), BTP is more like a general positive attitude towards time. All BTP indicators measured with ZTPI, including DBTP, are devoted to assess a healthy profile of TPs (Stolarski et al., 2020). The question is which time dimensions are used to measure BTP? From early studies, TP is defined as “the totality of one's psychological past and future” (Lewin, 1951). Therefore, Webster (2011) defined BTP as individuals' tendency to frequently and positively think about both their past and future and developed a scale including Past Orientation and Future Orientation to measure BTP. However, Webster (2011) focuses on only positive temporal dimensions, which is a simplistic view of the past and future since individuals are very likely to both positively and negatively think about past and future events. The positive views of the past and future can be regarded as protective factors for people's fulfilling and happy life, whereas negative views of the past and future act as risk factors for people's mental health (Boniwell et al., 2010; Carelli et al., 2015; Diaconu‐Gherasim et al., 2021; Zhang et al., 2013; Zimbardo & Boyd, 2008). Thus, we believe both positive and negative past and future dimensions are indispensable when measuring temporal balance.

More importantly, remembering the past and looking into the future take place in the present and one can only experience happiness in the present, which may make it the core factor of BTP (Zimbardo & Boyd, 2008). However, the ZTPI does not contain a clearly positive present dimension. The PH subscale of the ZTPI may not have unequivocally positive implications for well‐being indicators. For instance, PH was found to be positively related to positive affect in the study by Boniwell et al. (2010), but negatively related to happiness in the study by Drake et al. (2008). The reason for these inconsistent results may be that PH in ZTPI is measuring both impulsivity and enjoyment of life (Jankowski et al., 2020; Zimbardo & Boyd, 1999). We suggest that removing the measure of impulsivity and retaining the measure of enjoyment of present life could help clarify the relationship between PH and well‐being indicators. Although ZTPI also measures PF, we do not recommend measuring this dimension in the present TPs. PF is more likely to measure fatalism, lack of control over life, and sense of helplessness rather than TP (Webster, 2011; Worrell et al., 2018). Some items of PF such as “You can't really plan for the future because things change so much” and “It doesn't make sense to worry about the future, since there is nothing that I can do about it anyway” could concern future perspective, thus this dimension might not be necessary if there is a well‐constructed future subscale. Researchers have suggested that living positively and fully in the present is vital for a self‐updating person (Shostrom, 1974). Seema and Sircova (2013) proposed that mindfulness is both a TP and awareness of one's TPs. Vowinckel et al. (2015) developed a Present‐Eudaimonic Scale based on the concept of mindfulness and found it explains incremental validity in predicting mental health over the other dimensions of TP. Based on these ideas and results, we propose that the extent to mindfully live in the present can be regarded as the key component of BTP. Mindfulness refers to a non‐judging, present‐centered mode of awareness involving that awareness of one's thoughts and feelings occurring in the present moment (Bishop et al., 2004), which can be regarded as a particular way relating to the present, giving people opportunities to fully and positively live in the here and now (Rönnlund et al., 2019). In addition, according to Zimbardo's views, being hedonistic during the holiday is an adaptive way to relieve stress and maintain temporal balance, while being completely task‐focused and unwilling to spend time relating to family and friends might hinder temporal balance (Zimbardo & Boyd, 1999, 2008). In today's highly competitive society, many people may have high temporal stress and anxiety, and be in a state of endless work agendas instead of enjoying the present with family or friends. Such an excessively task‐oriented perspective, rejecting any personal indulgence, may fuel high stress levels, leading to poor mental health. Although excessively task‐oriented individuals may achieve career success, this may often lead to unhappiness in life (Zimbardo & Boyd, 1999). Therefore, it is also necessary to measure people's current behavioral orientation of overvaluing work or tasks. Taken together, mindful present (MP), PH, and present excessively task‐oriented (PET) might be used to represent present TP factors when assessing temporal balance.

Given that one can cognitively and emotionally review the past and look into the future, but can take action only in the present, we suggest using one's overall positive attitudes toward the past and future and the extent to which adaptive behavioral tendencies are exhibited in the present to structure the temporal balance. Individuals can be seen as having temporal balance when their level of positive views is high enough to moderate or counterbalance the adverse effects of negative TPs on subjective well‐being. Thus, a very simple method to determine an individual's temporal balance is to see whether they score higher on positive TPs than negative TPs. Regarding calculating BTP, we propose to use a Differentials Method (DM) by subtracting the standardized negative TP scores from the standardized positive TP scores. Before using the DM, the linear association between TPs and subjective well‐being needs to be satisfied. This premise can ensure that the increase in positive TPs or the decrease in negative TPs will lead to the corresponding increase or decrease of subjective well‐being rather than the opposite.

### The present studies

In the literature on TPs, ZTPI is the most widely used scale. However, the reliability and structural validity of ZTPI continues to be questioned (Davis & Cernas Ortiz, 2017; Mohammed & Marhefka, 2020). DBTP is a more efficient way to measure BTP and is linked to various aspects of human functioning, but such a method is likely to be maladaptive (Stolarski et al., 2020). DBTP measures the deviation from unrealistic psychological reality that few people can achieve, which may be not conducive to the advance of BTP (McKay et al., 2022). To make contributions to solving these problems to a certain extent, we try to develop a new scale with valid psychometric properties and provide a simple and useful method to calculate BTP.

The dimensions used to reflect BTP were initially set to seven factors including PP, PN, MP, PH, PET, future positive (FP), and future negative (FN). Given the pronounced effects of BTP on subjective well‐being (Stolarski et al., 2020), well‐being related to positive (life satisfaction and positive affect) and negative variables (negative affect and anxiety) were mainly used to examine the external validity of TPI in the present study. In this paper, we focus on the following research question: (1) Whether a seven‐factor model of TPI is suitable to measure BTP; (2) testing whether the DM can be used to calculate the BTP indicator, and examining its associations with subjective well‐being related variables; and (3) whether TPI is better than ZTPI in explaining subjective well‐being indicators. Two studies were conducted. Study 1 was designed to develop TPI and examine its psychometric properties. Study 2 re‐examined the validity of TPI by comparing the variances of subjective well‐being indicators and anxiety explained by TPI and the Chinese version of ZTPI.

## STUDY 1: DEVELOPMENT OF TPI

### Method

#### Participants and procedures

Two independent samples were recruited in Study 1 using an online platform (wjx.cn) and randomly distributed questionnaire links in the participant pool. Informed consent was obtained from participants and our study was approved by the ethics committee of the Faculty of Psychology at Southwest University. Participants were excluded if they randomly responded (Participants who gave the same answers for all items) or responded too quickly (Participants who responded in less than 180 s). This included 23 and 54 invalid participants in sample 1 and sample 2, respectively. After excluding invalid cases, sample 1 consisted of 529 participants ranging from 17 to 60 years old (64.7% females; *M* = 25.99, *SD* = 8.01). In sample 1, 3.6% reported they had not completed high school, 7.9% completed high school, 9.1% had vocational/junior college, 53.1% completed college, and 26.3% received post‐graduate degrees. Participants from sample 1 completed the initial TPI and were used for exploratory factor analysis. Sample 2 consisted of 577 participants ranging from 16 to 60 years old (77.9% females; *M* = 27.69, *SD* = 11.76). In sample 2, 4.3% had not completed high school, 10.4% completed high school, 11.1% had vocational/junior college, 71.4% received college degrees, and 2.8% received post‐graduate degrees. The data from sample 2 was used for confirmatory factor analysis and to examine the reliability and validity of TPI. A total of 105 participants from sample 2 completed the TPI twice at 4 weeks apart to examine test‐retest reliability.

#### Measures

##### TP inventory (TPI)

According to the TP theories and existing scales (Carelli et al., 2015; Sobol‐Kwapińska & Jankowski, 2016; Vowinckel et al., 2015; Zimbardo & Boyd, 1999) and expert feedback, we defined seven components of TPI: PP, PN, FP, FN, MP, PH, and PET. Items for the TPI were generated from interviews with experts and professors in the field and a critical review of existing measures of TPs. We identified initially 62 items by the process of selecting, modifying, and rebuilding. The seven‐factor model was used to guide the applicability of the generated items, and items that did not conform to the theoretical setting were deleted. A consultation was conducted on the appropriateness of the items generated for each dimension in an expert panel including 16 researchers in the field. Thirteen items were identified as potentially problematic and were deleted at the expert review stage, thus the preliminary version of TPI comprised 49 items rated on a 1 (*strongly disagree*)–5 (*strongly agree*) scale.

##### Perceived stress

The four‐item version of perceived stress scale (Leung et al., 2010) was rated on a five‐point Likert scale: *Never* (0), *almost never* (1), *sometimes* (2), *often* (3), and *very often* (4). Higher total scores indicate higher level of perceived stress. In this study, McDonald's ω score for perceived stress was .72.

##### Anxiety

The generalized anxiety disorder ‐7 scale was used (Spitzer et al., 2006). All items were rated on a four‐point Likert scale indicating symptom frequency, ranging from 0 (*not at all*) to 3 (*nearly every day*). Higher scores indicate higher levels of anxiety symptoms (ω = .92).

##### Positive and negative affect scale

The Chinese version of Positive and Negative Affect Scale (Qiu et al., 2008) has 18 items and two subscales: Positive Affect and Negative Affect. Participants were asked to rate the items from 1 (*very slightly or not at all*) to 5 (*extremely*). Higher scores indicate greater levels of positive affect (*ω* = .95) and negative affect (*ω* = .88).

##### Life satisfaction

Satisfaction with Life Scale (Diener et al., 1985; Xiong & Xu, 2009) has five items rated on a 1 (*strongly disagree*)–7 (*strongly agree*) scale. Higher scores reflect increased satisfaction towards life (*ω* = .91).

#### Data analyses

##### Exploratory factor analysis (EFA)

EFA with principal component analysis and varimax rotation was used to explore the factor structure of TPI. The adequacy of sampling was assessed by significant Bartlett's test of sphericity and the Kaiser–Meyer–Olkin value (KMO > 0.6) (Tabachnick & Fidell, 2013). Extracting and retaining factors were based on criteria such as eigenvalues >1 and the Scree Test. TP theory and the designed seven‐factor model were also used to determine the retention of items.

##### Confirmatory factor analysis (CFA)

CFA was used to further test the factorial structure of TPI. Given that scores for many items in sample 2 were not normally distributed (Shapiro–Wilk values ranging from .80 to .90), we used the robust maximum likelihood (MLR) estimation in CFAs (Satorra & Bentler, 1994). The comparative fit index (CFI) (≥ .95 for good, ≥ .90 for acceptable), Tucker‐Lewis Index (TLI) (≥ .95 for good, ≥ .90 for acceptable), root mean square error of approximation (RMSEA) (≤ .06 for good, ≤ .08 for acceptable), and standardized root mean square residual (SRMR) (≤ .08 for acceptable) were used to evaluate global model fit. We examined three models for different factor structures of TPI. The single‐factor model had each indicator in each construct load onto a single factor. The correlated multifactor model consisted of specific factors, which then correlated with one another. In the higher‐order model, each factor of TPI regresses onto a general higher‐order latent variable.

Internal consistency was assessed using McDonald's ω coefficients, and test–retest reliability was evaluated by Pearson's correlations. Criterion‐related validity was tested by the correlations between the TPI scores and subjective well‐being indicators, anxiety, and perceived stress. Based on the notion of BTP and findings of meta‐analyses (Diaconu‐Gherasim et al., 2021; Stolarski et al., 2020), we predicted that PP, MP, FP, PH, and BTP would be positively related to positive affect and life satisfaction, while PN, FN, and PET would be positively associated with negative affect, anxiety, and perceived stress. Results consistent with these predictions would support the criterion‐related validity of TPI.

### Results

#### EFA

The KMO value was .90 and Bartlett's test was significant (*χ* ^*2*^ = 13892.623, *df* = 1128, *p* < .001), indicating the 49 items were appropriate for factor analysis. Prior to EFA, the correlation matrix of all items was inspected. Five items were not significantly correlated with most of the other items and were excluded from further analyses. Regarding item selection, the remaining items were evaluated for deletion against the following criteria: (1) Items that had factor loadings below .45; (2) items that had two or more factor loadings; (3) the loading of an item on a factor that did not align with theory; (4) items that belonged to a factor that had three or fewer items. The use of these criteria resulted in a final set of 28 items that did not meet any of the criteria mentioned above.

The final EFA showed that seven factors had eigenvalues greater than 1 and explained 66.65% of the total variance. The factor loadings of the final 28 items are presented in Table 1. Each factor had four items and the resulting 28‐item TPI contained seven dimensions: (1) FP, reflecting a hopeful and optimistic attitude toward the future; (2) PN, involving a pessimistic and negative attitude towards the past; (3) FN, representing a hopeless and worrying attitude towards the future; (4) PP, which describes a positive and joyful outlook on the past; (5) PET, reflecting the extent to work or stay task‐focused and unwillingness to spend time relating to family or friends; (6) PH, involving a hedonic tendency with minimal concern with future consequences; (7) MP, describing a preference to mindfully live in the here and now and awareness of the value of each moment of life.

**TABLE 1:** Results of the exploratory factor analysis.

Items		Factor loadings						
		1	2	3	4	5	6	7
Future positive	T38	**.78**	−.05	−.22	.13	−.08	.02	.18
	T39	**.79**	−.05	−.27	.11	−.04	.03	.26
	T40	**.79**	−.06	−.22	.14	−.04	−.03	.25
	T42	**.61**	−.03	.06	.10	.05	.09	−.01
Past negative	T21	−.09	**.85**	.14	.08	.13	.04	−.02
	T22	.12	**.74**	.24	−.09	.06	.05	.02
	T24	−.11	**.80**	.16	−.01	.16	.22	.02
	T25	−.11	**.77**	.20	−.09	.19	.15	−.09
Future negative	T44	−.19	.21	**.70**	.04	.19	.13	−.05
	T45	−.22	.21	**.85**	.07	.08	.04	−.06
	T46	−.17	.24	**.83**	.01	.11	.05	−.13
	T47	.00	.14	**.64**	−.13	.08	.19	−.18
Past positive	T15	−.01	−.19	−.03	**.83**	.03	.06	.06
	T16	.07	−.08	.00	**.85**	−.02	.10	.03
	T19	.27	.08	.00	**.73**	.10	−.05	.22
	T20	.23	.14	.01	**.76**	.03	.00	.14
Present excessively task‐oriented	T2	−.09	.12	−.07	.04	**.73**	.18	.00
	T3	−.05	.24	.03	.04	**.73**	.04	.05
	T5	.02	.08	.22	.02	**.79**	−.01	−.03
	T7	.08	.04	.23	.02	**.78**	−.02	.02
Present hedonistic	T10	.08	.11	.09	.06	−.08	**.80**	−.08
	T11	−.03	.03	.00	−.04	−.06	**.75**	.27
	T13	.07	.19	.17	.05	.13	**.71**	−.11
	T14	−.04	.07	.09	.04	.19	**.70**	−.01
Mindful present	T26	.05	.03	−.17	.08	.01	.10	**.80**
	T27	.22	−.03	−.07	.17	−.02	.06	**.77**
	T30	.39	−.05	−.11	.13	.03	−.09	**.67**
	T31	.38	−.03	−.07	.12	.06	−.15	**.50**
Variance explained (%)		21.78	14.95	7.66	7.42	5.98	4.57	4.29
Cumulative variance explained (%)		21.78	36.72	44.38	51.81	57.79	62.36	66.65

#### Model testing

The single factor model had a poor fit to the data, *χ* ^*2*^(350) = 3660.26, *p* < .001, CFI = .398, TLI = .394, RMSEA = .128, SRMR = .133. The seven‐factor model had satisfactory fit indices: *χ* ^*2*^(328) = 631.632, *p* < .001, CFI = .945, TLI = .936, RMSEA = .040, SRMR = .059, and all indicators loaded on their factors at values of .30 and above. Regarding correlations, PP, PN, MP, FP, and FN were significantly related to each other (see Table 2 for more details). In these correlations, the highest coefficient is the correlation between MP and FP (*r* = .55), which echoes the finding of Wittmann et al. (2014) showing that mindfulness is related to a pronounced future perspective. In addition, PH and PET had small or non‐significant correlations with the other five factors except the moderate positive relationship between PET and PN and FN (see Table 2).

**TABLE 2:** Descriptive statistics, reliabilities, and correlations among subscales of time perspective inventory (TPI).

Dimensions of TPI	*M*	SD	*α*	*ω*	Test–retest	1	2	3	4	5	6
1 Past positive	3.33	.82	.88	.88	.70	1					
2 Past negative	2.62	.85	.86	.86	.74	−.14*	1				
3 Mindful present	3.71	.56	.78	.78	.52	.39**	−.18**	1			
4 Future positive	3.63	.67	.79	.82	.71	.37**	−.23**	.55**	1		
5 Future negative	2.38	.76	.84	.85	.63	−.22**	.40**	−.36**	−.47**	1	
6 Present excessively task‐oriented	2.87	.70	.73	.73	.78	−.02	.38**	−.06	−.05	.22**	1
7 Present hedonistic	3.10	.74	.73	.74	.79	.06	.03	.05	.12**	.11*	−.17**

* *p* < .05.

** *p* < .01.

**FIGURE 1:**
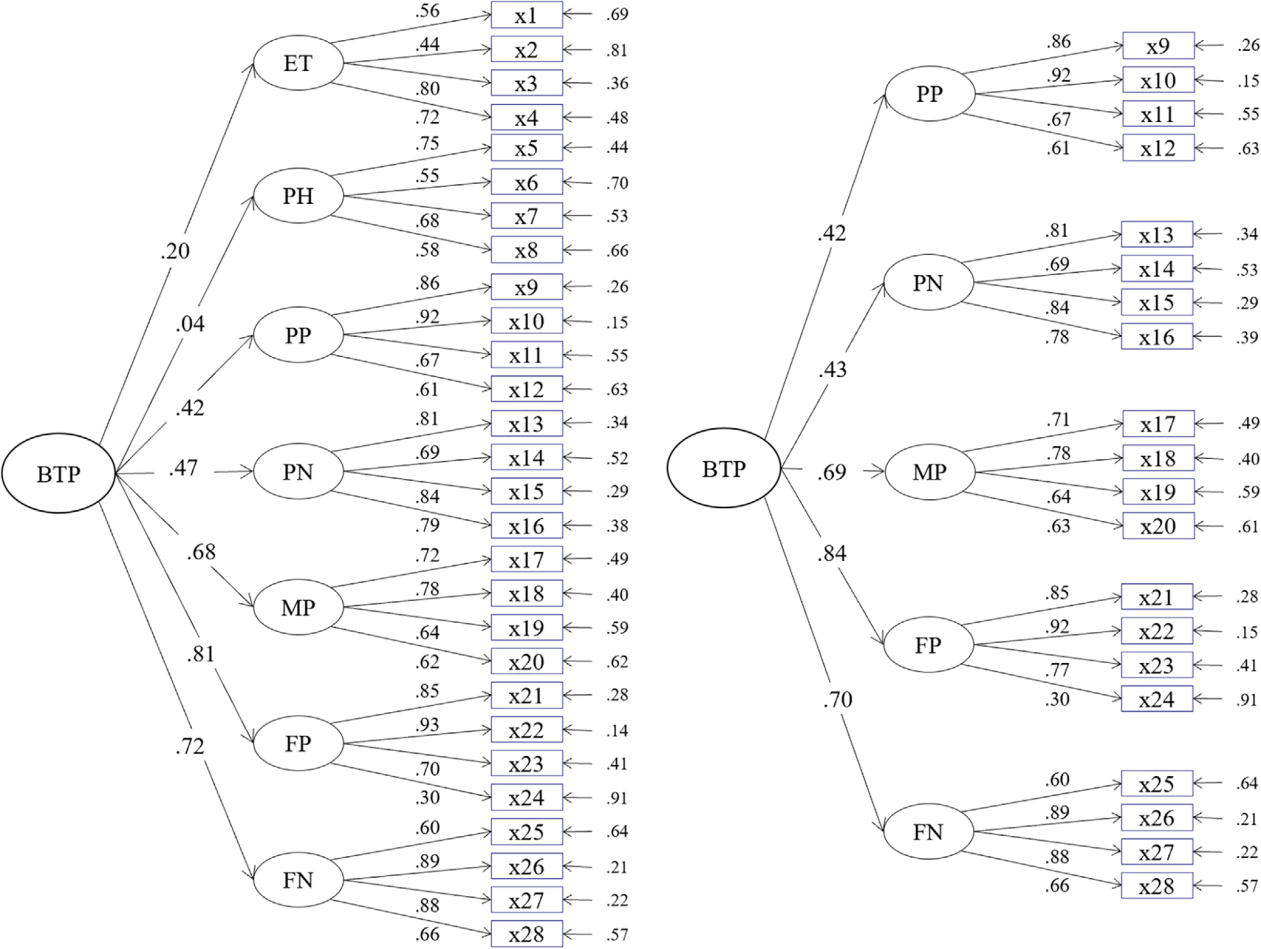
Factor loadings for the higher‐order model based on 7 factors (left) and 5 factors (right). PET, present excessively task‐oriented; FN, future negative; FP, future positive; MP, mindful present; PH, present hedonistic; PN, past negative; PP, past positive.

TPI both have positive and negative factors that are not conducive to the fitting of these factors to a high‐order factor. PN, FN, and PET are theoretically maladaptive for one's BTP, thus item scores from the three factors were reversed in the higher‐order model. Although the higher‐order model had an acceptable fit to the data (*χ* ^*2*^(342) = 758.14, *p* < .001, CFI = .924, TLI = .916, RMSEA = .046, SRMR = .080), the loadings of PH and PET on the higher‐order factor were not significant or below .30 (Figure 1).

After deleting these two factors, the correlated five‐factor model (*χ* ^*2*^(159) = 293.85, *p* < .001, CFI = .968, TLI = .962, RMSEA = .038, SRMR = .053) and higher‐order model (*χ* ^*2*^(164) = 327.84, *p* < .001, CFI = .961, TLI = .955, RMSEA = .042, SRMR = .065) both had a desirable model fit. All five factors on the higher‐order factor were statistically significant, ranging from .42 to .84 (Figure 1). This result may indicate that these five factors are more suitable to create a total composite score of BTP. Given that being moderately hedonistic instead of focusing excessively on tasks and goals are theoretically important components of a BTP (Zimbardo & Boyd, 1999, 2008), we retained PH and PET in the TPI. The final version of TPI and English items are shown in Appendix A.

#### Reliabilities

Based on sample 2, the results of reliability analyses indicated that all TPI subscales had adequate internal consistencies with ω coefficients ranging from .73 to .88. All test–retest reliability coefficients were strong, ranging from .52 to .79 (Table 2).

#### Examining the linear and curvilinear relationships between TPs and subjective well‐being

Multiple regressions were used to test the differences in the TPs in well‐being indicators. In all regression models, TPI factors and their square scores were entered as predictors, subjective well‐being indicators were entered as dependent variables, respectively. Quadratic trends in six out of 21 regression models were significant (Table 3). However, all these quadratic associations followed a U shape, indicating that these optimal levels would be below the mean TP scores or outside the range of actual TP scores. For example, PP and positive affect are U‐relationship patterns, but the increase of PP scores from 1 to 5 would increase positive affect. Such a result may indicate that PP predicts positive affect in a linear manner. Coupled with the fact that most of the quadratic trends of TPs and well‐being indicators were not significant, we regard the relationships between TPs and well‐being as a linear trend. According to the direction of the relationships between seven factors and subjective well‐being, PP, FP, MP, and PH can be regarded as positive TPs, and PN, FN, and PET can be regarded as negative TPs. Thus, the DM can be used to calculate BTP, which means that BTP = *z*‐scores on positive TPs (PP, FP, MP, PH) – *z*‐scores on negative TPs (PET, PN and FN). A higher score on BTP indicates a better level of overall temporal balance.

**TABLE 3:** Regression results of time perspectives on indicators of subjective well‐being.

Model	Predictors	Life satisfaction			Positive affect			Negative affect		
		*β*	*t*	*R* ^*2*^ adj	*β*	*t*	*R* ^*2*^ adj	*β*	*t*	*R* ^*2*^ adj
1	Intercept	2.13		.129	2.21		.128	2.22		.006
	PP	.37***	7.68		.29***	6.90		−.06	−1.25	
	PP squared	.08	1.67		.12*	2.93		.06	1.25	
2	Intercept	5.45		.085	4.40		.158	.89		.196
	PN	−.27***	−5.75		−.33***	−8.34		.41***	10.73	
	PN squared	−.04	−.89		.07	1.70		.11**	2.81	
3	Intercept	.83		.195	.61		.257	3.15		.057
	MP	.44***	9.54		.48***	12.33		−.24***	−5.38	
	MP squared	−.01	−.14		.07	2.03		.04	.94	
4	Intercept	.90		.318	1.42		.225	3.00		.115
	FP	.55***	13.41		.43***	11.13		−.30***	−7.17	
	FP squared	−.03	−.63		.05	1.38		.13**	3.25	
5	Intercept	5.84		.161	4.55		.196	1.05		.194
	FN	−.41***	−8.50		−.41***	−9.97		.38***	9.20	
	FN squared	.05	.99		.13**	3.18		.14**	3.48	
6	Intercept	4.99		.036	4.32		.122	1.00		.127
	PET	−.16**	−3.43		−.27***	−6.66		.33***	8.40	
	PET squared	.06	1.18		.030	.76		.09*	2.27	
7	Intercept	3.47		.028	3.01		.060	2.42		.008
	PH	.13**	2.79		.09*	2.17		−.10*	−2.4	
	PH squared	−.04	−.92		.01	.12		−.01	−.32	

*Note*: Control variables were gender, age, and education level.

Abbreviations: PET, present excessively task‐oriented; FN, future negative; FP, future positive; MP, mindful present; PH, present hedonistic; PN, past negative; PP, past positive.

* *p* < .05.

** *p* < .01.

*** *p* < .001.

#### Criterion‐related validity

As shown in Table 4, PP had a positive relationship with life satisfaction and positive affect, and a negative relationship with perceived stress. MP and FP were positively related to life satisfaction and positive affect, and negatively related to negative affect, anxiety, and perceived stress. PN, FN, and PET were negatively related to life satisfaction and positive affect, and positively related to negative affect, anxiety, and perceived stress. PH was positively related to life satisfaction and positive affect, and negatively related to negative affect. These results provide evidence for the criterion‐related validity of TPI. Based on the seven‐factor model of TPI, we calculated two indicators of temporal balance including BTP calculated by the DM and DBTP calculated by the DBTP formula (the optimal value of PP, FP, MP, and PH was set to 5, and the optimal value of PET, PN, and FN was set to 1). Results showed that BTP was negatively correlated to DBTP (*r* = −.96, *p* < .001). The BTP was found to be positively related to life satisfaction and positive affect and negatively related to negative affect, perceived stress, and anxiety. The opposite pattern was found for DBTP. The correlation coefficients of BTP and DBTP with criterion variables were comparable. These findings support the effectiveness of the DM to calculate BTP.

**TABLE 4:** Criterion‐related validities of time perspective inventory.

Variables	Life satisfaction	Positive affect	Negative affect	Anxiety	Perceived stress
Past positive	.36**	.30**	−.07	.01	−.25**
Past negative	−.27**	−.32**	.43**	.53**	.47**
Mindful present	.45**	.49**	−.23**	−.19**	−.32**
Future positive	.57**	.44**	−.30**	−.30**	−.37**
Future negative	−.40**	−.40**	.41**	.43**	.47**
Excessively task‐oriented	−.15**	−.26**	.35**	.40**	.29**
Present hedonistic	.15**	.12**	−.09*	−.03	.08
BTP‐DM	.61**	.59**	−.47**	−.48**	−.54**
BTP‐DBTP	−.57**	−.53**	.47**	.48**	.55**

Abbreviations: BTP‐DM, indicator of balanced time perspective calculated by DM; DBTP, indicator of balanced time perspective calculated by DBTP formula.

* *p* < 0.05.

** *p* < 0.01.

## STUDY 2: RETEST THE EFFECTIVENESS OF TPI

Study 2 aimed to retest the effectiveness of TPI by comparing the variances of subjective well‐being indicators and anxiety explained by TPI and the Chinese version of ZTPI.

### Method

#### Participants and procedures

Participants in study 2 were also recruited via wjx.cn. Data of 58 participants were excluded due to invalid responses (e.g., too‐short answering time and the same answers for all items). After excluding invalid data, the final sample in Study 2 consisted of 713 participants ranging from 17 to 79 years old (78.1% females; *M* = 40.15, *SD* = 15.73). Regarding education, 6.6% received post‐graduate degrees, 36.5% had a college degree, 18.7% graduated from vocational/junior college, 23.3% graduated from high school, and 15.0% had not completed high school. In terms of family income, 27.2% of participants reported that their annual household income was less than 30,000 RMB, 41.4% were between 30,000 and 80,000 RMB, 21.3% were between 80,000 and 150,000 RMB, and 10.1% were between 150,000 and 800,000 RMB. The investigation obtained approval from the corresponding author's University Ethics Committee. All participants consented to participate in this study and finishing the questionnaire took about 10–20 min.

#### Measures

##### TP inventory (TPI)

All participants completed the TPI developed in Study 1. In the present sample, McDonald's ω values were .87 for PP, .86 for PN, .83 for FP, .85 for FN, .84 for MP, .74 for PET, and .77 for PH. The BTP was calculated using the DM.

##### Chinese version of Zimbardo TP Inventory (ZTPI‐C)

This 25‐item scale revised by Li et al. (2022) has five dimensions: PN, PP, F, Present Impulsive (PI), and PF. Participants were asked to rate items from 1 (*strongly disagree*) to 5 (*strongly agree*). In this study, ω scores of the five subscales were .88, .85, .83, .74, and .76, respectively. The DBTP was calculated to represent the indicator of time balance. Jankowski et al. (2020) propose using extreme values and they changed the optimal level of TPs into PN 1, PP 5, PF 1, PH 3.4, and F 5. In ZTPI‐C, PH was revised into PI which is characterized by carelessness and disregard for consequences. PI in ZTPI‐C is clearly maladaptive, thus we used 1 as its optimal value. Higher scores on DBTP reflect the lower temporal balance.

##### Anxiety

The same anxiety scale as in study 1 was used to measure participants' generalized anxiety (*ω* = .91).

##### Subjective well‐being indicators

Positive and negative affect and satisfaction with life scales consistent with Study 1 were used as indicators of subjective wellbeing (Busseri & Sadava, 2011). In the present study, ω scores for positive affect, negative affect, and life satisfaction were .93, .90, and .90, respectively.

#### Data analyses

Correlation analysis was preliminarily used to examine the relationships among study variables. Hierarchical regression was used to compare the relations of BTP (measured with TPI) and DBTP (measured with ZTPI‐C) and subjective well‐being indicators and anxiety.

### Results

#### Correlations

As shown in Table 5, BTP measured with TPI was significantly associated with DBTP measured with ZTPI‐C, supporting the congruent validity of TPI. BTP measured with TPI was found to be positively associated with life satisfaction and positive affect and negatively related to negative affect and anxiety. The opposite pattern was found for DBTP measured with ZTPI‐C.

**TABLE 5:** Means, standard deviations, and correlations among study variables.

Variables	*M*	SD	1	2	3	4	5
1 BTP measured with TPI	.18	3.40	1				
2 DBTP measured with ZTPI‐C	4.19	.78	−.43**	1			
3 Anxiety	12.86	3.83	−.44**	.43**	1		
4 Life satisfaction	4.30	1.18	.46**	−.30**	−.25**	1	
5 Positive affect	2.64	.77	.35**	−.24**	−.20**	.37**	1
6 Negative affect	1.95	.67	−.41**	.38**	.60**	−.20**	−.03

Abbreviations: BTP, balanced time perspective; DBTP, deviation from the balanced time perspective; TPI, time perspective inventory.

** *p* < .01.

#### The link of BTP and DBTP with subjective well‐being indicators and anxiety

In all hierarchical regressions, gender, age, education level, and annual household income were entered as predictors in step 1, followed by the DBTP measured with ZTPI‐C or BTP measured with TPI entered in step 2, whereas subjective well‐being indicators and anxiety were entered as dependent variables. As shown in Table 6, the regression coefficients and explained variance of BTP measured with TPI on life satisfaction and positive affect were all higher than DBTP measured with ZTPI‐C. The explained variance of BTP measured with TPI on negative affect and anxiety were all relatively higher than DBTP measured with ZTPI‐C, but the regression coefficients of BTP measured with TPI and DBTP measured with ZTPI‐C on negative affect and anxiety were comparable.

**TABLE 6:** The regression results of BTP and DBTP on well‐being indicators and anxiety.

	Life satisfaction		Positive affect		Negative affect		Anxiety	
Predictors	*β* (t)	*β* (t)	*β* (t)	*β* (t)	*β* (t)	*β* (t)	*β* (t)	*β* (t)
Gender	.05 (1.34)	.02 (.68)	.01 (.36)	.01 (.14)	−.04 (1.04)	−.02 (−.58)	.04 (1.16)	.06 (1.66)
Age	.16** (2.93)	.19*** (3.93)	−.16** (3.01)	−.12* (−2.41)	−.03 (−.64)	−.10* (−2.03)	−.05 (−.91)	−.13** (−2.59)
Education level	−.04 (−.72)	.02 (.46)	−.06 (−1.08)	−.01 (−.10)	.09 (1.67)	.02 (.29)	.19*** (3.51)	.10 (1.89)
Annual household income	.13** (3.22)	.08* (2.17)	.14** (3.34)	.11** (2.62)	−.05 (1.23)	−.03 (−.68)	−.06 (−1.64)	−.04 (−1.03)
DBTP measured with ZTPI‐C	−.26*** (−7.15)		−.24** (−6.59)		.37*** (10.40)		.42*** (12.47)	
BTP measured with TPI		.44*** (12.98)		.34*** (9.67)		−.39*** (−11.29)		−.43*** (−12.76)
*F*	19.75***	44.65***	15.12***	25.57***	26.39***	30.34***	42.74***	44.31***
Adjusted *R* ^2^	.116	.235	.090	.147	.157	.171	.227	.233
△*R* ^2^	.064	.181	.056	.112	.129	.149	.169	.176

*Note*: Standardized coefficients from step 2 of all models are shown in the table for clarity and simplicity.

Abbreviations: BTP, balanced time perspective; DBTP, deviation from the balanced time perspective; TPI, time perspective inventory.

* *p* < .05.

** *p* < .01.

*** *p* < .001.

## DISCUSSION

We sought to develop and test the psychometric properties of a comprehensive measure of the overall TP: The TPI. The TPI was designed to assess temporal balance across three time frames (past, present, and future) of attitudes and behavioral tendencies. Although there is a widely used ZTPI to measure TPs and calculate the temporal balance, it has been problematic with poor reliabilities, mismatch between items and dimensions, and lack of important components that reflect BTP, such as a positive present, a negative future, and an excessive task orientation (Carelli et al., 2015; McKay et al., 2015; Webster, 2011; Worrell et al., 2018; Zimbardo & Boyd, 1999). The TPI was designed to address these gaps.

### Reliability and validity of TPI

The results from Study 1 demonstrated that the seven‐factor of TPI produced reliable subscales and a valid measure of TPs. Four of the seven factors, including PP, PN, FP, and FN, assess the positive and negative attitudes an individual holds toward the past and future. The present TP is a psychological bridge to connect the past and the future. The other three factors of TPI, including MP, PH, and PET, assess one's behavioral tendencies in the present. MP was conceptualized to capture a tendency to fully and attentively live in the here and now, which is evidently beneficial to positive emotions and life satisfaction (Sobol‐Kwapińska, 2013). PH assesses the extent to focus on immediate pleasures without concern for future consequences. Focusing on immediate pleasures may lead to maladaptive behaviors like procrastination, but not enjoying the present life may also lead to accumulated physical and mental stress and poor mental health. PET assessed the extent to which one was focused on work or present tasks with no willingness to spend time relating to family or friends. In today's global economy, those excessively task‐oriented people surrounded by endless work agendas may have a high level of stress and poor mental health and may need therapy to develop a broader TP (Zimbardo & Boyd, 1999). No scales in previous studies are designed for measuring excessive task orientation, and we added it in TPI and regard it as an important component that impacts whether an individual has BTP.

CFAs showed that the correlated seven‐factor model and the higher‐order model of TPI had acceptable fitness. However, PH and PET factors were weakly related to the other factors and their loadings on BTP were not significant or had low coefficients. After excluding these two factors, both the correlated five‐factor model and the higher‐order model had desirable fitness. These results may indicate that the overall orientation toward the past and the future combined with mindfully living in the present are more suitable statistically to measure BTP. Given that emphasizing immediate pleasures in the present instead of focusing on tasks and goals excessively are theoretically important components of a BTP (Zimbardo & Boyd, 1999, 2008), PH and PET were also retained and used to calculate BTP in this study.

The TPI had good internal consistencies, and Cronbach's alpha coefficients (.73–.88) were higher than many other versions of the ZTPI (McKay et al., 2015; Worrell et al., 2018). Comparatively, the α values of TPI were also greater than the Chinese versions of ZTPI, for instance, the α values for Chinese versions of ZTPI range from .57 to .75 (Li et al., 2022) and .32 to .66 (Wang et al., 2015), respectively. In addition, the high coefficients of the four‐week test–retest reliability supported the temporal stability of the TPI scores. Also, seven subscale scores correlated with subjective well‐being indicators, anxiety, and perceived stress, and the direction of these relationships was consistent with previous studies (Diaconu‐Gherasim et al., 2021; Mooney et al., 2017; Rönnlund et al., 2019). These results support the reliability and validity of TPI.

### The effectiveness of the DM to calculate BTP

We propose to use the DM to calculate BTP by adopting the standardized scores of positive TPs minus the standardized scores of negative TPs. Before using this method, we tested the linear and quadratic relationships between TPs and subjective well‐being. Regression results in study 1 indicated linear relationships between all seven factors of TPI and subjective well‐being indicators, which were consistent with previous studies (Jankowski et al., 2020). Based on the seven‐factor model of TPI, the correlation coefficient (*r* = −.96) between indicators of BTP calculated by the DM and the DBTP formula was almost approaching 1. The BTP calculated by the DM had moderate to large relationships with subjective well‐being indicators, perceived stress, and anxiety, which also supported the effectiveness of the DM.

### The relationships between TPI and indicators of subjective well‐being

To further examine the utility of TPI, we compared the links between BTP measured with TPI and DBTP measured with the ZTPI‐C and well‐being indicators and anxiety in study 2. The results showed that the variance of subjective well‐being indicators and anxiety explained by BTP were all higher than DBTP. Specifically, the regression coefficients of BTP on life satisfaction and positive affect were higher than DBTP, while the regression coefficients of DBTP and BTP on negative affect and anxiety were comparable. These results showed that TPI may be better in explaining positive outcomes since we theoretically put more emphasis on the positive views toward time and added a positive present perspective which is crucial to one's well‐being (Vowinckel et al., 2015). The reason why DBTP can also better explain the variation of negative affect and anxiety may be that the ZTPI‐C contains more negative TP factors including PN, PI, and PF (Li et al., 2022). PI in ZTPI‐C was formed by items reflecting impulsiveness from PH in ZTPI (Li et al., 2022). Thus, it can be seen as a maladaptive factor because impulsiveness is negatively associated with self‐control and subjective well‐being (Barker et al., 2015; Love & Holder, 2014). Considering that impulsivity is not appropriate to reflect PH (Worrell et al., 2018), similar items from ZTPI referring to impulsiveness or risk‐taking were not included in TPI. PF was a significant risk factor for one's subjective well‐being (Diaconu‐Gherasim et al., 2021), but was not considered in TPI in this study, because it is more likely to measure fatalism, lack of control over life, and a sense of helplessness rather than TP. Taken together, the seven‐factor TPI may be more suitable for Chinese adults to measure a BTP.

### Contributions, limitations, and future directions

This study makes two main contributions. Firstly, it is the first study to develop a scale measuring overall temporal balance in China. Compared with ZTPI‐C (Li et al., 2022), TPI has good reliability and factor structure. Different from the previous scales designed for teenagers and college students (Chan et al., 2019; Lyu & Huang, 2016; Worrell et al., 2013), we developed TPI based on larger age samples ranging from 16 to 79 years old, which may be more conducive to future exploration of the life course of TPs. In addition, TPS is half the length of the ZTPI and might have an advantage in avoiding the problem of respondent fatigue. Secondly, we provide a new approach to calculating BTP, namely the DM, through which we can effectively establish the relationships between BTP and subjective well‐being indicators. Compared to ZTPI, the DM is simpler and easier to use and is compatible with our concept of BTP. Different from the deviation from an unrealistic psychological reality (McKay et al., 2022), the DM sets that people have temporal balance when their positive perspectives are higher than negative ones. Therefore, we believe that TPI and the DM may facilitate future studies exploring the relations between individual differences in BTP and aspects of human functioning.

This study has some shortcomings. Firstly, the reliance on self‐reported measures may inflate the relationship among study variables. Using various sources to collect data is a way for future research to improve the objectivity of the study. Secondly, the association between PH and subjective well‐being needs to be further examined in future studies. PH in the TPI, which does not measure impulsiveness, mainly assesses the extent to enjoy life in the present, thus it may benefit for current life satisfaction and boost happiness. However, only focusing on immediate pleasures may lead to maladaptive behaviors like procrastination. It would be valuable to investigate whether PH has an adverse effect on subjective well‐being through procrastination. Thirdly, we should note that using the DM to calculate the total BTP score may weaken the effects of specific grouping factors since findings from the higher‐order model of TPI in study 1 indicated that the general BTP factor more represents the common variations of PP, PN, MP, FP, and FN factors. It would be valuable to investigate whether PH and PET have roles in temporal balance for special groups, such as employees in organizations. Being hedonistic could relieve stress and contribute to life satisfaction, but it may also lead to maladaptive behaviors and hinder one's success (Boniwell et al., 2010; Keough et al., 1999). In today's global economy, being excessively task‐oriented may accelerate career success, but being surrounded by endless work agendas could cause high stress and poor mental health (Zimbardo & Boyd, 1999). Thus, the trade‐off between these two orientations may play an important role in the balance of people's career success and happiness. Fourthly, the original definition of BTP is related to switching capacity between TPs (Zimbardo & Boyd, 1999), but we did not intend to assess the switching ability in the present studies because an overall switching capacity score may not be useful for measuring an individual's specific patterns of TPs. Future research may benefit from creating novel TP concepts, scales, and experimental procedures to assess the switching capacity between TPs in response to situational demands. Fifthly, the cross‐sectional design of our two studies limits conclusions about the relationships between BTP and subjective well‐being‐related outcomes. Longitudinal studies are needed to examine the linear or quadratic relationships between factors of TPI and subjective well‐being and to explore the developmental trajectory of BTP and its covariant relations with subjective well‐being‐related outcomes. In addition, given that the pronounced effects of temporal balance on subjective well‐being have been demonstrated (Stolarski et al., 2020), more attention should be paid to the mechanisms responsible for such an association. Finally, given that the TPI being developed and verified uses samples of Chinese adults, whether it applies to teenagers and samples from other cultural contexts needs to be tested in the future.

## CONCLUSIONS

In conclusion, the seven‐factor model of TPI showed good psychometric properties, including a stable factor structure, adequate internal consistencies, and satisfactory test–retest reliability. We found the hypothesized correlations between seven‐factor TPI and well‐being‐related outcomes. Furthermore, the TPI explained noteworthy variances of subjective well‐being indicators, above and beyond that of the DBTP measured with ZTPI. Taken together, the TPI appears to be a reliable and valid measurement to assess overall temporal balance.

## CONFLICT OF INTEREST STATEMENT

The authors declare that they have no conflicts of interest.

## ETHICS STATEMENT

This study was approved by the Institutional Review Board of the Faculty of Psychology, Southwest University. The participants signed an informed consent form stating the aim of the study and explaining that they could withdraw from the study, and the data would be anonymous.

## Data Availability

The datasets generated for this study are available on reasonable request from the corresponding author.

## APPENDIX A

**Table:**

ITEMS OF THE TIME PERSPECTIVE INVENTORY	
	Chinese (English)
Past positive	1 对我来说回忆过去是件快乐的事情 (Remembering the past is a happy thing for me)
	2 回忆过去增加了我生活中的乐趣 (Thinking about the past gives me pleasure in my life)
	3 重温以前的生活让我有了人生的方向感 (Reminiscing about my past life gives me a sense of direction)
	4 追忆过去给了我生活的目标感 (Reviewing events from my past gives me a sense of purpose in life)
Past negative	5 过去的痛苦经历在我的脑海中反复出现 (Painful past experiences keep being replayed in my mind)
	6 过去有太多不愉快的记忆, 我不愿去想 (The past has too many unpleasant memories that I prefer not to think about)
	7 我常想起那些曾经发生在我身上的坏事情 (I think about the bad things that have happened to me in the past.)
	8 我的过去让我难过 (My past makes me sad)
Mindful present	9 当我专注于当前的事物时, 我感到内心的平静与和谐 (I feel a certain peace and harmony when I stay focused on the present)
	10 专注于此时此刻发生的事情, 让我充满活力 (I feel revitalized after staying focused on the present)
	11 我能把注意力集中在此时此刻发生的事情上 (I'm able to focus on what's happening here and now)
	12 每一天, 我都试着让自己的生活变得充实 (I try to live my life as fully as possible, one day at a time)
Present hedonistic	13 我喜欢及时行乐, 明天的事明天再想 (I like to enjoy the pleasures of life here and now without thinking about tomorrow)
	14 对于我来说, 生命重在享受过程而不是结果 (It is more important for me to enjoy life's journey than to focus only on the destination)
	15 我只会采取行动来满足当前的需要, 至于以后的事, 以后再说 (I only act to satisfy immediate concerns, figuring the future will take care of itself)
	16 我只想做自己喜欢做的事情, 而不是逼迫自己按时完成某件事或某个任务 (I just want to do what I love to do instead of pushing myself to finish something or a task on time)
Future positive	17 我对自己的未来充满乐观 (I am optimistic about the future)
	18 我的将来充满希望 (Anticipating my future life fills me with hope)
	19 我对我的将来很憧憬 (I'm very excited about my future)
	20 在不确定的情况下, 我常常期望最好的结果 (In uncertain situations, I usually expect the best)
Future negative	21 思考我的将来让我不开心 (Thinking about my future makes me sad)
	22 我觉得自己的未来是毫无希望的 (I feel that my future is hopeless)
	23 我觉得自己的未来是没有前途的 (I feel that my future is unpromising)
	24 我不想思考我的未来, 因为思考未来没有意义 (I don't like to think about my future because it is meaninglessness)
Present excessively task‐oriented	25 我从未感到自己的时间充裕 (I never feel like I have enough time)
	26 稍微浪费一点时间都能让我很焦虑 (Wasting of time makes me anxious)
	27 我的生活中总是被工作或任务填满, 很少去享受生活 (My life is full of work or tasks that I rarely enjoy life)
	28 我很少花时间与家人、朋友在一起玩乐, 因为我有很多工作或任务要做 (I seldom spend time with my family and friends because I have too much work or tasks to do)

*Note*: Each item should be rated on a 5‐point scale: 1 = *strongly disagree*, 2 = *disagree*, 3 = *neither agree or disagree*, 4 = *agree*, 5 = *strongly agree*.
